# The Tomato U-Box Type E3 Ligase PUB13 Acts With Group III Ubiquitin E2 Enzymes to Modulate FLS2-Mediated Immune Signaling

**DOI:** 10.3389/fpls.2018.00615

**Published:** 2018-05-08

**Authors:** Bangjun Zhou, Lirong Zeng

**Affiliations:** Department of Plant Pathology, Center for Plant Science Innovation, University of Nebraska, Lincoln, NE, United States

**Keywords:** tomato, ubiquitin, U-box, PUB13, pattern-triggered immunity, FLS2, ubiquitin-conjugating enzyme (E2), immune signaling

## Abstract

In Arabidopsis and rice, the ubiquitin ligase PUB13*-*mediated protein degradation plays a significant role in plant pattern-triggered immunity (PTI) and flowering time control. The Arabidopsis PUB13 has been shown to attenuate the pattern recognition receptor FLS2-mediated immune signaling by ubiquitinating FLS2 and consequently promoting its degradation by the 26S proteasome. Nevertheless, the cognate ubiquitin-conjugating enzymes (E2) with which PUB13 acts to modulate FLS2-mediated PTI are unknown. To address this question, we investigate here the tomato (*Solanum lycopersicum*) homolog of PUB13, SlPUB13 by utilizing the recently characterized complete set of tomato E2s. Of the 13 groups of tomato E2s, only members in group III are found to interact and act with SlPUB13. Knocking-down of the group III E2 genes enhances callose deposition and induction of the *RbohB* gene in the immunity-associated, early oxidative burst after flg22 treatment. The group III E2s are also found to work with SlPUB13 to ubiquitinate FLS2 *in vitro* and are required for PUB13-mediated degradation of FLS2 *in vivo* upon flg22 treatment, suggesting an essential role for group III E2s in the modulation of FLS2-mediated immune signaling by PUB13. Additionally, another immunity-associated E3, NtCMPG1 is shown to also work specifically with members of group III E2 in the *in vitro* ubiquitination assay, which implies the group III E2 enzymes may cooperate with many E3 ligases to regulate different aspects of PTI. Taken together, these data corroborate the notion that group III E2 enzymes play an important role in PTI and build a foundation for further functional and mechanistic characterization of tomato PUB13.

## Introduction

The plant immune system conceptually consists of two layers of active defense responses, microbe/pathogen -associated molecular pattern (MAMP/PAMP)-triggered immunity (MTI/PTI) and effector-triggered immunity (ETI) (Jones and Dangl, 2006; Macho and Zipfel, 2014; Cui et al., 2015). PTI is initiated upon perceiving PAMPs by plant pattern recognition receptors (PRR) (Jones and Dangl, 2006; Coll et al., 2011; Shamrai, 2014). Activation of PTI triggers an array of defense responses including production of reactive oxygen species (ROS), modulation of defense-related gene expression, and callose deposition at the cell wall (Hann and Rathjen, 2007; Nguyen et al., 2010; Veluchamy et al., 2014). PTI as the first line of immune responses is important for fending off most potential plant pathogens. To promote colonization, pathogens deploy various effectors into the host cell to suppress or evade PTI (Rosebrock et al., 2007). However, some of these effectors are detected by intracellular nucleotide-binding leucine-rich repeat (NLR) proteins thus activate the second layer of defense, ETI, which is usually accompanied by programed cell death (PCD) at the site of infection that restricts the spreading of pathogen (Jones and Dangl, 2006).

Several PAMPs and their corresponding PRRs have been identified and studied (Felix et al., 1999; Kunze et al., 2004; Kaku et al., 2006; Kawaharada et al., 2015), among which the bacterial flagellin and flg22, a 22-amino acid, immunogenic fragment of flagellin and their plant receptor, Flagellin Sensing 2 (FLS2) have been well characterized and are considered as a model for functional and mechanistic studies of PTI (Gomez-Gomez and Boller, 2000; Lu et al., 2011). The Arabidopsis FLS2 cooperates with the co-receptor, BRI1-Associated Receptor Kinase 1 (BAK1) to sense bacterial flagellin/flg22 and initiates immune signaling (Chinchilla et al., 2007). To prevent excessive or prolonged activation of immune responses, flagellin induces the recruitment of two closely related U-box type ubiquitin ligases (E3) PUB12 and PUB13 to the Arabidopsis FLS2 receptor complex to ubiquitinate and consequently promote degradation of FLS2 by the 26S proteasome (Lu et al., 2011). Upon flg22 treatment, Arabidopsis *pub12* and *pub13* mutants displayed increased ROS production and callose deposition and elevated induction of immune responsive genes than the wild type plants (Lu et al., 2011). The *pub13* mutant also displays early flowering, spontaneous cell death, accumulation of hydrogen peroxide and salicylic acid (SA), and elevated resistance to biotrophic pathogens under long-day (LD) condition in a SID2- and PAD4-dependent manner, which implies that PUB13 plays dual roles in the regulation of both plant defense and development via SA-mediated signaling (Li et al., 2012a). In rice, the *Spotted Leaf11* (*Spl11*) encodes a U-box type E3 ubiquitin ligase and is the ortholog to the Arabidopsis PUB12 and PUB13 (Zeng et al., 2002, 2004). The rice loss-of-function mutant *spl11* displays broad-spectrum resistance to rice bacterial and fungal pathogens (Yin et al., 2000). A further investigation indicated the Rho GTPase-activating protein, SPIN6 (SPL11-interacting Protein 6) interacts with SPL11 and OsRac1 and negatively regulates programmed cell death and innate immunity in rice (Liu et al., 2015). Unlike Arabidopsis mutant *pub13* with an early-flowering phenotype under LD conditions, the rice mutant *spl11* displays a delayed-flowering phenotype under LD conditions (Vega-Sanchez et al., 2008), suggesting opposite functions of Arabidopsis PUB13/SPL11 in flowering time control. However, overexpression of *Spl11* can complement the cell death and flowering phenotype of the *pub13* mutant (Li et al., 2012b), which indicates the roles of PUB13/SPL11 in the control of flowering time and in defense are conserved in monocots and dicots. In Arabidopsis, a LysM receptor kinase, Chitin Elicitor Receptor Kinase 1 (CERK1) is essential for the chitin elicitor-triggered immune signaling (Miya et al., 2007). Chitin-induced formation of a complex of Lysin Motif Receptor Kinase5 (LYK5) and CERK1 leads to activation of the CERK1 intracellular kinase domain and induction of plant innate immunity in Arabidopsis (Cao et al., 2014). Recently, it was reported that PUB13 regulates the abundance of chitin receptor LYK5 protein (Liao et al., 2017). Additionally, PUB13 was also found to regulate the abundance of the ABA co-receptor ABI1 (Kong et al., 2015) and SA-mediated induction of pathogenesis-related gene expression (Antignani et al., 2015). These data indicate PUB13 plays an important role in multiple immune signaling pathways in Arabidopsis and rice. Nevertheless, identification and functional characterization of the tomato homolog of PUB13 have not been reported so far.

Ubiquitination as a major post-translational protein modification in eukaryotic cells typically entails a stepwise enzymatic cascade that is catalyzed by three different classes of enzymes, the ubiquitin-activating enzyme (E1), the ubiquitin-conjugating enzyme (E2), and the ubiquitin ligase (E3) (Zhou and Zeng, 2017). During the ubiquitination process, the E2∼ubiquitin intermediate cooperates with an E3 to transfer ubiquitin to the substrate. In the past decade, research has centered on ubiquitin E3 ligases due to their key role in determining the substrate specificity in ubiquitination. By contrast, ubiquitin-conjugating enzymes (E2) were often considered as “carrier of ubiquitin” with an auxiliary role in the ubiquitination process. However, emerging evidence indicates E2 enzymes are the key mediators of chain assembly (Ye and Rape, 2009). In addition, our recent studies implicated the tomato E2 enzymes Fni3 and the group III E2s in plant immunity (Mural et al., 2013; Zhou et al., 2017). The group III E2 enzymes were found to be essential for plant PTI and for the suppression of host immunity by the ubiquitin ligase activity of a *Pseudomonas syringae* pv. *tomato* (Pst) effector, AvrPtoB (Zhou et al., 2017; Zhou and Zeng, 2017). Additionally, functional redundancy among group III members was revealed for their role in PTI and in AvrPtoB-mediated suppression of plant immunity. In previous studies that demonstrated the *in vitro* ubiquitination of FLS2 (Lu et al., 2011), ABI1 (Kong et al., 2015) and LYK5 (Liao et al., 2017) by PUB13, the Arabidopsis UBC8, a member of the group III E2s was used for the assays. However, whether other E2 enzymes in the genome can also work with PUB13 is unclear.

In this study, we identify and characterize the tomato closest homolog of PUB13, SlPUB13 in plant immunity. Our results indicate that SlPUB13 works specifically with members of group III tomato E2 enzymes for the ubiquitination of FLS2 in modulating FLS2-mediated immune signaling. These data corroborate the notion that group III E2 enzymes play an important role in PTI.

## Materials and Methods

### Growth of Bacteria and Plant Materials

*Agrobacterium tumefaciens* strains GV3101 and GV2260 and *Pseudomonas fluorescens* 55 were grown at 28°C on Luria-Bertani and King’s B medium, respectively with appropriate antibiotics. *Nicotiana benthamiana* and tomato RG-pto11 (*pto11/pto11, Prf/Prf*) seeds were germinated and plants were grown on autoclaved soil in a growth chamber with 16 h light (∼300 μmol/m^2^/s at the leaf surface of the plants), 24°C/23°C day/night temperature, and 50% relative humidity.

### DNA Manipulations and Plasmid Constructions

All DNA manipulations were performed using standard techniques (Sambrook and Russell, 2001). The opening reading frame (ORF) and U-box domain of tomato *SlPUB13* gene, *NtCMPG1* and kinase domain of *FLS2* were amplified using the Q5 High-Fidelity DNA Polymerase (New England Biolabs) and then cloned into the pENTR/SD/D-TOPO Gateway entry vector by following the protocols provided by the manufacturer (Life Technologies). *SlPUB13* and *NtCMPG1* in the pENTR/SD/D-TOPO vector were transferred to the pDEST15 vector using LR reaction according to instructions provided by the manufacturer (Life Technologies) for expression and purification of GST-SlPUB13 and GST-NtCMPG1. *SlPUB13-U-box* and the twelve group III E2 genes (Zhou et al., 2017) in the pENTR/SD/D-TOPO vector was cloned into the pNLexAattR or pJZ4attR (Finley et al., 2002) vector using LR reaction according to instructions provided by the manufacturer (Life Technologies) for yeast two-hybrid assay. *FLS2-KD* was digested with the restriction enzymes *Eco*RI and *Xho*I and cloned into the protein expression vector pMAL-c2 that was restricted by enzymes *Eco*RI and *Sal*I. The constructs used for BiFC assay were prepared by digesting the vectors pA7-nYFP and pA7-cYFP with the restriction enzymes *Xho*I and *Sma*I, followed by ligation of the corresponding gene that has been amplified using the Q5 High-Fidelity DNA Polymerase (with an adapted 5′ *Xho*I restriction site but without the stop codon) as described previously (Zhou et al., 2017). To monitor the degradation of the kinase domain SlFLS2-KD of SlFLS2 in the presence of PUB13 in *N. benthamiana* protoplasts, the genes *Fni3C89G* and *SlFLS2-KD* were cloned into a pTEX 35S cauliflower mosaic virus promoter expression cassette with HA tag at the C terminus (Mural et al., 2013). Primers used for recombinant DNA cloning are listed in **Supplementary Table S1**.

### Sequence Alignment and Phylogenetic Analysis

For sequence alignment, sequences of interest in the FASTA format were input into the ClustalX 2.1 program and aligned using the ClustalX algorithm (Larkin et al., 2007). The phylogenetic analysis was then performed with the MEGA6 program using the aligned sequences (Tamura et al., 2013) as described previously (Zhou et al., 2017). The accession numbers of the Arabidopsis PUB proteins are available at the Arabidopsis Information Resource (TAIR) website^1^ (Azevedo et al., 2001).

### Yeast Two-Hybrid Assay

The LexA-based yeast two-hybrid system, the corresponding procedures for testing protein-protein interaction, and the detection of bait and prey proteins by immunoblot were as described previously (Mural et al., 2013).

### Bimolecular Fluorescence Complementation (BiFC) Assay

The BiFC assay based on split YFP was used to test the interaction of E2 proteins and SlPUB13 in *N. benthamiana* leaf protoplasts (Chen et al., 2006; Waadt et al., 2008; Zhou et al., 2017). The non-group III E2 UBC13 fused to the N-terminus of YFP and the empty vectors expressing the N-terminus and C-terminus of YFP (nYFP-EV and cYFP-EV) were used as negative control. Protoplasts were prepared from leaves of wild type *N. benthamiana* plants as described (Rosebrock et al., 2007). Approximately 1 × 10^4^ protoplasts that were suspended in a volume of 200 μL were then co-transfected with 10 μg plasmid DNA of each individual of the construct pair to be tested. The co-transfected protoplast was imaged 21 h after transfection using an Olympus FV500 Inverted (Olympus IX-81) Confocal Microscope with the following excitation (ex) and emission (em) wavelengths: YFP, 514.5 nm (ex), 525–555 nm (em); chlorophyll auto-fluorescence, 640.5 nm (ex) and 663–738 nm (em).

### Expression and Purification of Recombinant Proteins

GST- and MBP-tagged fusion proteins were expressed in *E. coli* strain BL21 (DE3) and purified with Glutathione Sepharose 4 Fast Flow beads (GE Healthcare) and Amylose Resin High Flow (New England Biolabs), respectively by following the protocol provided by the manufacturer. The purified proteins were further desalted and concentrated in the protein storage buffer (50 mM Tris-HCl pH8.0, 50 mM KCl, 0.1 mM EDTA, 1 mM DTT, 0.5 mM PMSF) using the Amicon Centrifugal Filter (Millipore). The desalted and concentrated recombinant protein was stored at -80°C in the presence of a final concentration of 40% glycerol. The concentration of purified protein was determined using protein assay agent (Bio-Rad). The quality of purified proteins was analyzed by 10% SDS-PAGE.

### *In Vitro* Ubiquitination Assay

The *in vitro* ubiquitination assays shown in **Figure 2** and **Figure 7** were performed together with the assay previously described (Figure 3 in Zhou et al., 2017) using the same protocol. To test the ubiquitination of FLS2 by PUB13 and group III E2, the following protocol was used. 3 μg ubiquitin, 40 ng E1 (GST-SlUBA1), optimal amount (50–250 ng) of E2 protein (GST-SlUBC8, GST-SlUBC10, GST-SlUBC27, or 6xHis-SlUBC12) (Zhou et al., 2017), 2 μg E3 ligase (GST-SlPUB13 or GST-NtCMPG1, **Supplementary Figure S2**) and 1 μg substrate (MBP-FLS2-KD or MBP) were added to a 30 μL reaction in the presence of ubiquitination assay buffer (50 mM Tris-HCl pH7.5, 5 mM ATP, 5 mM MgCl_2_, 2 mM DTT, 3 mM creatine phosphate, 5 μg/ml creatine phosphokinase). The reactions were incubated at 30°C for 1.5 h and then terminated by addition of SDS sample loading buffer with 100 mM DTT. The reaction products were resolved by 10% SDS-PAGE and analyzed by immunoblotting using mouse monoclonal anti-FLAG M2-peroxidase-conjugated (horseradish peroxidase) antibody (Sigma-Aldrich) for identifying the poly-FLAG-Ubiquitin signal. Polyubiquitinated forms of MBP-FLS2-KD were detected using anti-MBP antibody (New England Biolabs).

### Virus-Induced Gene Silencing (VIGS)

Silencing of group III E2 genes was induced using the tobacco rattle virus (TRV) vectors (Caplan and Dinesh-Kumar, 2006) as described (Zhou et al., 2017). The group III E2 genes-silenced plants and non-silenced control plants used for experiments shown in **Figure 5** were from the same batches of plants described previously (Zhou et al., 2017). The efficiency of silencing group III E2 genes was detected as previously shown (Supplementary Figure S9C in Zhou et al., 2017).

### Callose Deposition Assay

The measurement of callose deposition was performed as previously described (Nguyen et al., 2010) with minor modifications. Leaves of group III ubiquitin E2 genes-silenced (TRV-*group III*) and non-silenced TRV control *N. benthamiana* plants (3–4 weeks after VIGS infiltration) were infiltrated using 1 mL needleless syringe with 40 μM flg22, 40 μM flgII-28 or a suspension of *P. fluorescens* 55 at 2.5 × 10^8^ CFU/mL (OD_600_ = 0.5), respectively. Leaf disks of 10 mm in diameter were then excised using a cork borer from infiltrated areas 24 h after the infiltration, followed by incubation at 37°C in wells of a 12-well plate containing 2 mL 95% ethanol until the leaf disks were cleared of chlorophyll. The incubation time for clearing the chlorophyll of leaf was up to 48 h and the ethanol was replaced as necessary until the clearing process was complete. The cleared leaf disks were washed two times with 70% ethanol and then three times with distilled water. The leaf disks were immersed in 0.1% aniline blue in 150 mM K_2_HPO_4_ (pH 9.5/KOH) and incubated in the dark for 1 h. The stained leaf disks were mounted with 60% glycerol on glass slides and observed from the adaxial surface of the disk by a fluorescence microscope (Zeiss Axionplan 2, Carl Zeiss, Oberkochen, Germany). The number of deposited callose was counted using the ImageJ analysis software (Schindelin et al., 2012).

### Transient Expression of Recombinant Proteins in *N. benthamiana* Leaf Protoplasts

To monitor the degradation of tomato FLS2 cytoplasmic kinase domain (FLS2-KD) by endogenous PUB13, approximately 6 × 10^4^ protoplasts in a volume of 200 μL were prepared from leaves *N. benthamiana* plants, three to four weeks after VIGS infiltration. The protoplasts were then co-transfected with 7 μg of plasmid pTEX-*SlFLS2-KD-HA* and 5 μg of plasmid pTEX-*Fni3C89G-HA*. Seventeen hours after transfection the protoplasts were treated with 1 μM of flg22 for 30 min, followed by protein extraction and immunoblotting as described (Zhou et al., 2017). The total proteins were analyzed by immunoblot using anti-HA antibody.

### Real Time RT-PCR

To reveal the expression pattern of the plant immunity marker gene *RbohB*, leaves of the group III ubiquitin E2 genes-silenced (TRV-*group III*) and non-silenced TRV control *N. benthamiana* plants similar to those being used in the callose deposition assay were infiltrated with 40 μM flg22 using a needleless syringe. Leaf samples were harvested at 0 (before inoculation), 0.5, 1, 3, 6, 9, 10, 13, and 24 h post-infiltration. For all the samples harvested, extraction of total RNA, synthesis of first strand cDNA, and qRT-PCR were conducted as described previously (Zhou et al., 2017). *NbEF1α* was used as an internal reference. All primers used in qRT-PCR are showed in the **Supplementary Table S1**.

## Results

### Identification of the Closest Tomato Homolog of PUB12 and PUB13

**FIGURE 1:**
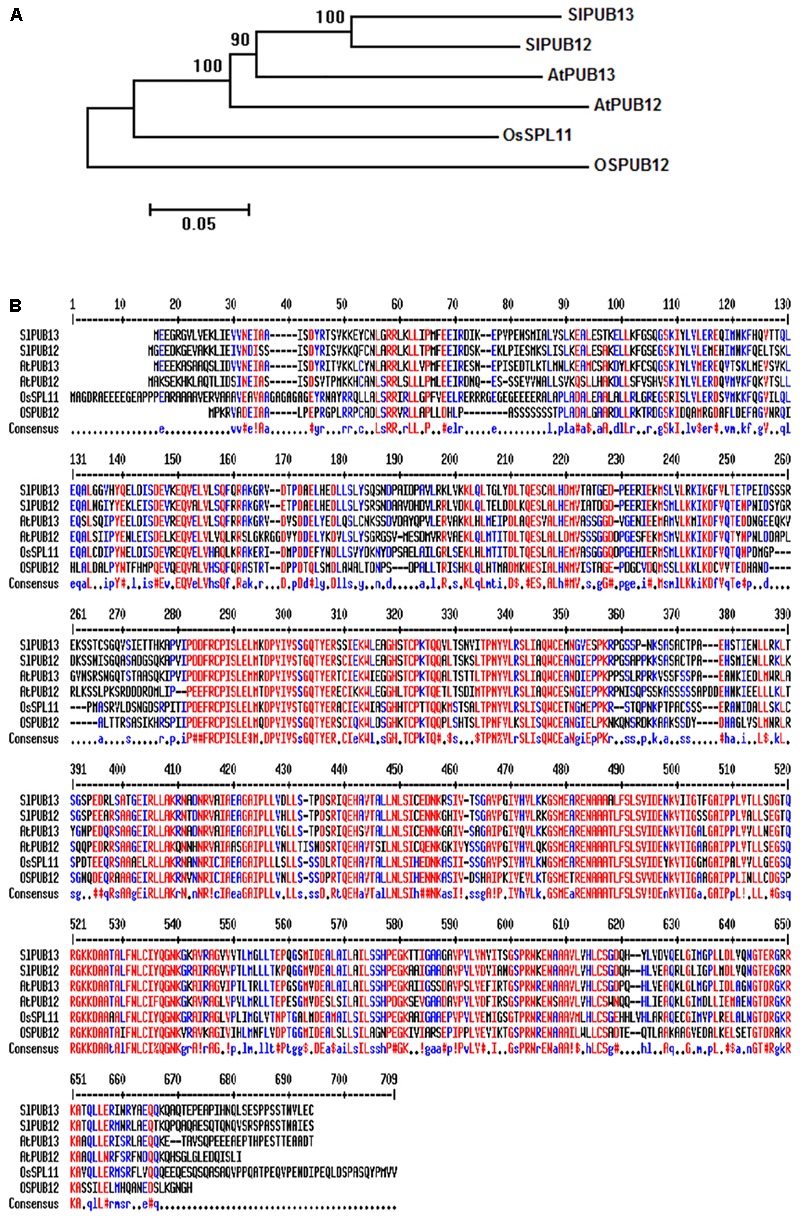
Phylogenetic analysis and sequence alignments of PUB12 and PUB13 homologs from tomato, Arabidopsis and rice. **(A)** Phylogenetic analysis of PUB12 and PUB13 from tomato, Arabidopsis and rice. The sequences of PUB12 and PUB13 proteins were used for generating the tree. The unrooted phylogenetic tree was generated by the neighbor-joining method using the MEGA6 program with 1000 bootstrap trials (Saitou and Nei, 1987; Tamura et al., 2013). **(B)** Sequence alignments of PUB12 and PUB13 from tomato, Arabidopsis and rice. The sequences of PUB12 and PUB13 proteins in the FASTA format were entered into MultAlin and aligned using the default parameters (Corpet, 1988). Color red denotes high consensus amino acids while blue and black denotes low and neutral consensus ones, respectively.

To study the function of tomato PUB13 and the E2 enzymes with which it works in plant immunity, we searched the tomato genome in the Sol Genomics Network (SGN^2^) database using the sequences of Arabidopsis PUB12 (AtPUB12) and PUB13 (AtPUB13) as query. Twenty-one tomato homologous proteins of AtPUB12 and AtPUB13 were identified. The tomato genes *Solyc11g066040.1* and *Solyc06g076040.2* that encode the closest homolog to AtPUB12 and AtPUB13, respectively were named *SlPUB12* and *SlPUB13* based on the phylogenetic analysis (**Supplementary Figure S1**) of the tomato homologs and the Arabidopsis PUB proteins (Azevedo et al., 2001). Phylogenetic analysis of the PUB12 and PUB13 homologs from tomato, Arabidopsis and rice indicated tomato PUB12 and PUB13 proteins (hereafter designed as SlPUB12 and SlPUB13, respectively) are phylogenetically closer to the counterparts from Arabidopsis (**Figure 1A**). Sequence alignments revealed the tomato, Arabidopsis and rice PUB12 and PUB13 proteins are highly homologous, with AtPUB13 and SlPUB13 are 66.1% identical whereas the SlPUB12 and SlPUB13 proteins are 78.2% identical in amino acid sequence (**Figure 1B**).

### SlPUB13 Works Specifically With the Tomato Group III E2s to Catalyze Ubiquitination

**FIGURE 2:**
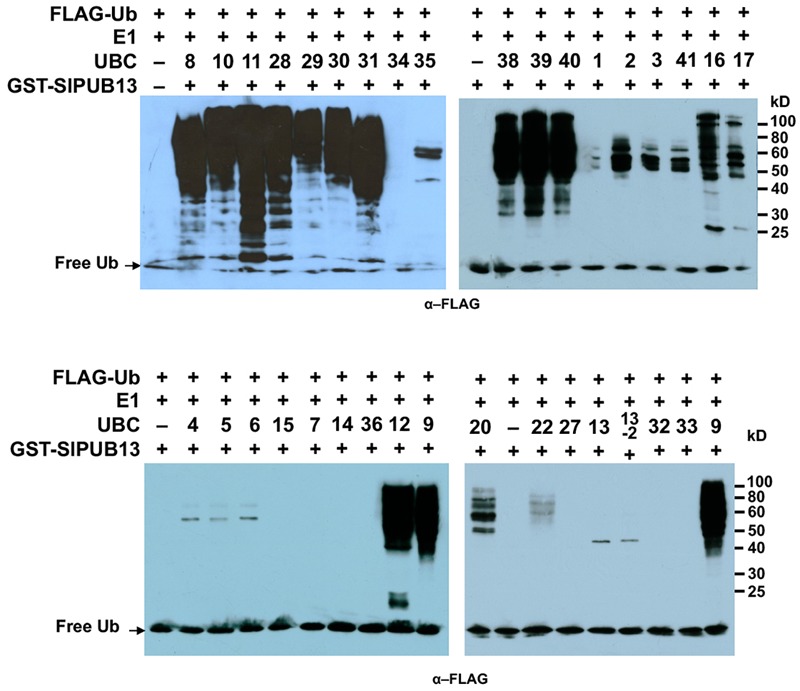
SlPUB13 shows specificity toward group III E2s in *in vitro* ubiquitination assays. GST-SlPUB13 was tested against 34 purified tomato E2 proteins (Zhou et al., 2017) in *in vitro* ubiquitination assays to determine its specificity toward ubiquitin E2s. Tomato E2 protein used in each reaction is shown above the lane by their UBC number and the minus marker (–) denotes the absence of any E2 enzyme in the reaction. The presence of high molecular weight (MW) (>98 kD, size of GST-SlPUB13) smear of ubiquitinated proteins as detected by Western blot using anti-FLAG antibody indicates E2-E3 specificity. The numbers on the right denote the molecular mass of marker proteins in kilodaltons. This experiment was repeated two times with similar results.

We previously identified and cloned a set of forty tomato genes that encode ubiquitin E2 proteins (Zhou et al., 2017). Of the 40 genes, we were able to purify the recombinant protein for 35 of them and demonstrate 34 of the purified E2 proteins possessed E2 ubiquitin-conjugating activity (Zhou et al., 2017). To find out which tomato E2 Enzymes work with SlPUB13, the E2-E3 specificity between SlPUB13 and the 34 enzymatically active E2s was examined by *in vitro* ubiquitination assay. To ensure the accuracy of the assay, we utilized SDS-PAGE to examine the quality of purified recombinant E2 proteins and the thioester assay to determine the amount of each E2 to be used for the assay so that similar amount of ubiquitin was activated and conjugated to each E2 (**Supplementary Figures S4, S5** in Zhou et al., 2017), which is critical for testing the E2-E3 specificity. The amount of each E2 protein determined in those experiments was used for testing the PUB13-E2 specificity. In *in vitro* ubiquitination assay RING and U-box type ubiquitin E3s work with their cognate E2 to produce self-ubiquitination (Lorick et al., 1999; Hatakeyama et al., 2001; Zeng et al., 2004). As shown in **Figure 2**, in the presence of tomato E1, FLAG-tagged ubiquitin, SlPUB13, and required co-factors, reactions that contain members of group III tomato E2 enzymes, UBC8, 9, 10, 11, 12, 28, 29, 30, 31, 38, 39, and 40, respectively produced strong poly-ubiquitin signal that was shown as high molecular weight (MW) smear. By contrast, weak signal with MW less than that of GST-SlPUB13 (98 kD) was detected in reactions that contain UBC1, 2, 3, 41, 7, 4, 5, 6, 13, 13-2, 20, 22, and 35, respectively, which suggests none of the signal is self-ubiquitination by SlPUB13. Comparison of the pattern of the signal in these reactions with our previous results of thioester assay for tomato E2s (Zhou et al., 2017) suggested that the signals detected in the *in vitro* ubiquitination assay for these 11 E2s were E2-ubiquitin adducts. Therefore, the tomato UBC1, 2, 3, 4, 7, 13, 13-2, 20, 22, and 35 did not work with SlPUB13 in catalyzing ubiquitination. This result is in consistence with previous studies that the Arabidopsis UBC8, a member of the group III E2s was used for examining the *in vitro* ubiquitination of FLS2, ABI1, and LYK5 by PUB13 (Lu et al., 2011; Liu et al., 2012; Kong et al., 2015). The signal detected in the reactions that contain tomato UBC16 and 17, respectively is weak but include high MW smear (**Figure 2**). However, the tomato UBC16 and 17 have been demonstrated to be capable of catalyzing the formation of poly-ubiquitin chains in the absence of an E3 (Zhou et al., 2017). The high MW smear in the reactions that contain tomato UBC16 and 17 may not be resulted from the action of UBC16 and 17 with SlPUB13. To confirm this, we performed *in vitro* ubiquitination assays in the presence of GST-SlPUB13 or GST. The 6HIS-UBC12 from group III was included as control (**Supplementary Figure S3**). In reactions where SlPUB13 was absent (lane 2 and 5), tomato UBC16 and 17 produced poly-ubiquitin and GST-UBC16- or 17-(Ub)n polyubiquitin ladders. The addition of GST-SlPUB13 or GST to the reaction both enhanced the signal but did not alter the pattern of the ladders (lane 1, 4, 7, and 8). These results indicate tomato UBC16 and 17 are capable of catalyzing poly-ubiquitination in the absence of an E3. Additionally, the GST-SlPUB13-(Ub)n signals were detected only in the reaction where the 6HIS-UBC12 of group III was presented (lane 10), which indicated the SlPUB13 does not work with UBC16 and 17 to catalyze self-ubiquitination. These data confirm SlPUB13 does not have specificity toward UBC16 and UBC17 but is able to promote their activity (**Supplementary Figure S3**) and the high MW smear signals in the reactions that contain tomato UBC16 and 17 (**Figure 2**) were not the product of SlPUB13 self-ubiquitination, which is similar to our previous finding (Zhou et al., 2017). Taken together, we conclude that only members of the tomato group III E2s work with SlPUB13 to catalyze ubiquitination.

### Members of Group III E2s Interact With Tomato PUB13 *in Vivo*

**FIGURE 3:**
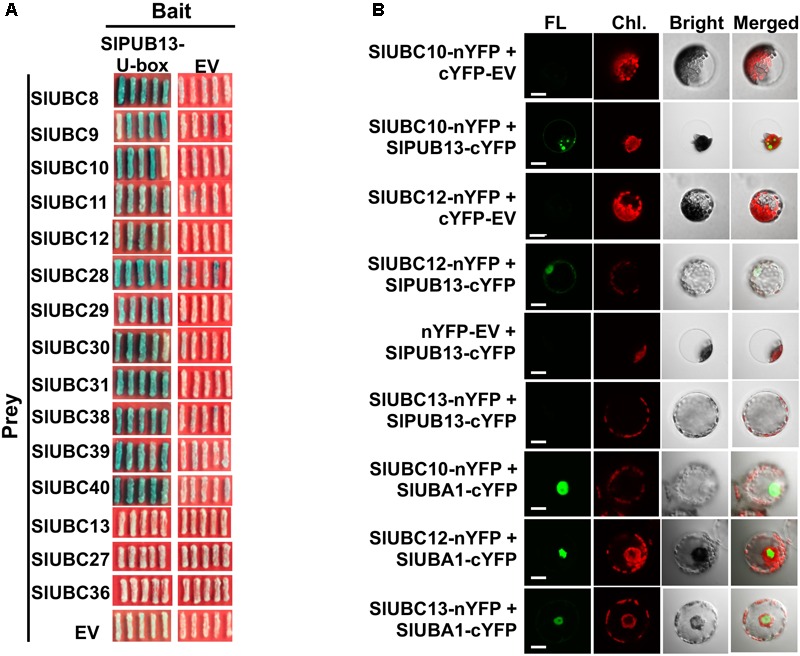
Group III E2s interact with tomato SlPUB13 *in vivo*. **(A)** Members of the group III E2s interact with the U-box domain of tomato SlPUB13 in yeast two-hybrid. Empty vectors and non-group III E2s were used as the negative control. **(B)** SlUBC10 and SlUBC12 of group III E2s interact with SlPUB13 in the BiFC assay. Non-group III E2 SlUBC13 was used as negative control. Different construct pairs were transiently co-expressed in protoplasts isolated from *N. benthamiana* leaves. Cells were examined with a confocal microscope under bright or laser light to detect cells and green fluorescence, respectively. The empty vector expressing N- and C-terminus of YFP (nYFP-EV and cYFP-EV) were used as negative control. EV, empty vector; FL, fluorescence; Chl., chlorophyll autofluorescence; Bright, bright field image. Scale bar = 20 μm.

To confirm the interaction of SlPUB13 with the group III E2s, yeast two-hybrid was employed to test the interaction of tomato group III ubiquitin E2 enzymes and the U-box domain of SlPUB13. Structural data available so far indicate that the RING (Really Interesting New Gene) and U-box domain of RING/U-box type E3s are responsible for the interaction with their cognate ubiquitin E2s in the ubiquitination process (Lorick et al., 1999; Zheng et al., 2000; Ohi et al., 2003; Christensen et al., 2007; Xu et al., 2008; Yin et al., 2009). We therefore tested the interaction of the U-box domain of SlPUB13 with group III E2 proteins by yeast two-hybrid. Non-group III E2 UBC13, 27, 36, 16, and 17 were used as control. As shown in **Figure 3A** and **Supplementary Figure S4A**, all group III E2 members interacted with SlPUB13 while the control and empty vector did not. Western blot confirmed the expression of the protein of the bait (SlPUB13 U-box) and the prey (i.e., the E2 proteins been tested) in the yeast cells (**Supplementary Figure S4B**). This result corroborates that only group III E2s but not E2s from other groups interact with SlPUB13 (**Supplementary Figure S4**). To further examine whether SlPUB13 interacts with the group III E2 proteins *in vivo*, BiFC assay was performed in *N. benthamiana* protoplasts. Tomato group III members UBC10 and UBC12 were randomly selected and non-group III E2 enzyme UBC13 was used as control for the assay (**Figure 3B**). As shown in **Figure 3B**, fluorescence signal was observed in protoplasts co-transfected with *UBC10-nYFP* and *PUB13-cYFP* or *UBC12-nYFP* and *PUB13-cYFP*, indicating that SlPUB13 interacted with UBC10 and UBC12 *in vivo*. By contrast, no fluorescence signal was observed in protoplasts co-transfected with *UBC10-nYFP* and *cYFP-EV* (*empty vector*), *UBC12-nYFP* and *cYFP-EV, UBC13*-*nYFP* and *PUB13-cYFP* or *nYFP-EV* and *PUB13-cYFP*. To ensure that the failure of UBC13 to interact with SlPUB13 was not due to problem with the *UBC13*-*nYFP* construct, the interaction of UBC10, 12 and 13 with the tomato E1, UBA1 were further tested using BiFC. As shown in **Figure 3B**, fluorescence was observed in protoplasts where the tomato E1 gene, *UBA1* was co-transfected with *UBC10, 12* and *13*, respectively, indicating the proteins of E1 and the E2s were expressed in the protoplasts and interacted as expected.

### Tomato PUB13 Works With Group III E2s to Ubiquitinate FLS2 *in Vitro*

**FIGURE 4:**
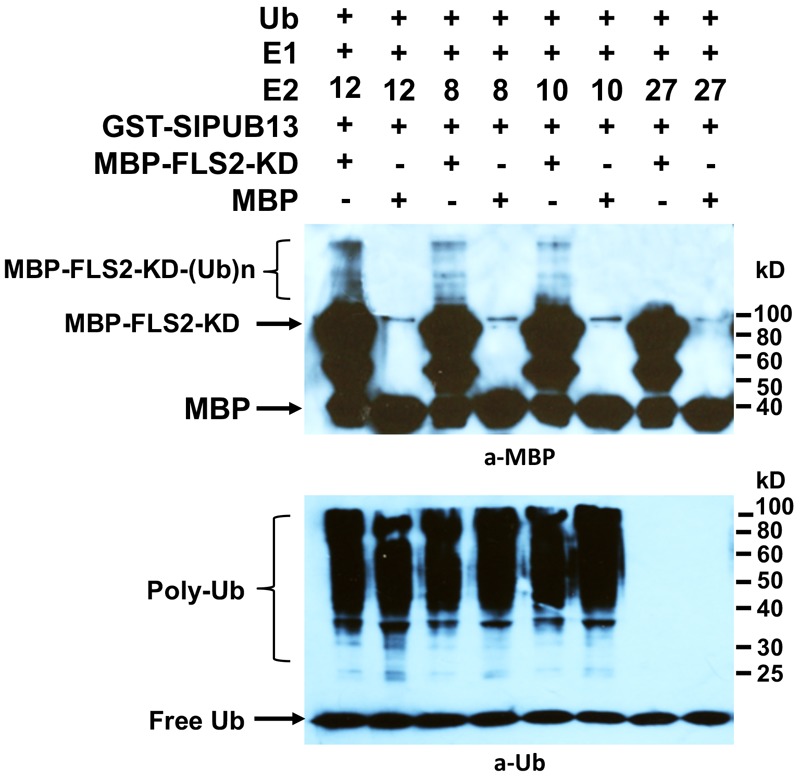
Tomato PUB13 works with group III E2s to ubiquitinate FLS2-KD. FLS2-KD was ubiquitinated in the presence of GST-SlPUB13 and a member of group III E2 in *in vitro* ubiquitination assay. The assay was performed with recombinant E1, E2, GST-SlPUB13, ubiquitin (Ub), and MBP-FLS2-KD. Reactions with non-group III E2 UBC27 were used as control. The MBP protein was used as control for the substrate. This experiment was repeated two times with similar result.

The Arabidopsis AtUBC8, a member of the group III E2s was shown to work with AtPUB13 in ubiquitinating FLS2 *in vitro* (Lu et al., 2011). Interestingly, our results indicated that only group III members of tomato E2s work with SlPUB13 to catalyze ubiquitination (**Figure 2**). It is thus intriguing to find out whether other members of tomato group III E2s work with SlPUB13 in the ubiquitination of the tomato FLS2. Similar to the previous study (Lu et al., 2011), we utilized the recombinant, MBP-tagged kinase-domain (MBP-FLS2-KD) of tomato FLS2 as the substrate for the *in vitro* ubiquitination assay. We used the MBP protein alone as the control. The results indicated that, in the presence of tomato E1, ubiquitin, SlPUB13, and required co-factors, MBP-FLS2-KD was ubiquitinated in reactions that contain group III E2 enzyme UBC8, 10 and 12, respectively but was not modified by ubiquitination in the reaction that contains the non-group III yet close-related outsider E2 UBC27 (Zhou et al., 2017) (**Figure 4**). The MBP protein alone was not ubiquitinated by SlPUB13 in reactions in which UBC8, 10 and 12 and 27 were presented, respectively. The presence of polyubiquitinated forms of FLS2-KD, MBP-FLS2-KD-(Ub)n in reactions where member of group III E2s is presented whereas lack of the signal in reactions where UBC27 is presented indicate SlPUB13 work specifically with group III E2s to ubiquinate FLS2-KD.

### Knocking Down Group III E2s Enhanced flg22-Induced Callose Deposition and Diminished Degradation of FLS2

**FIGURE 5:**
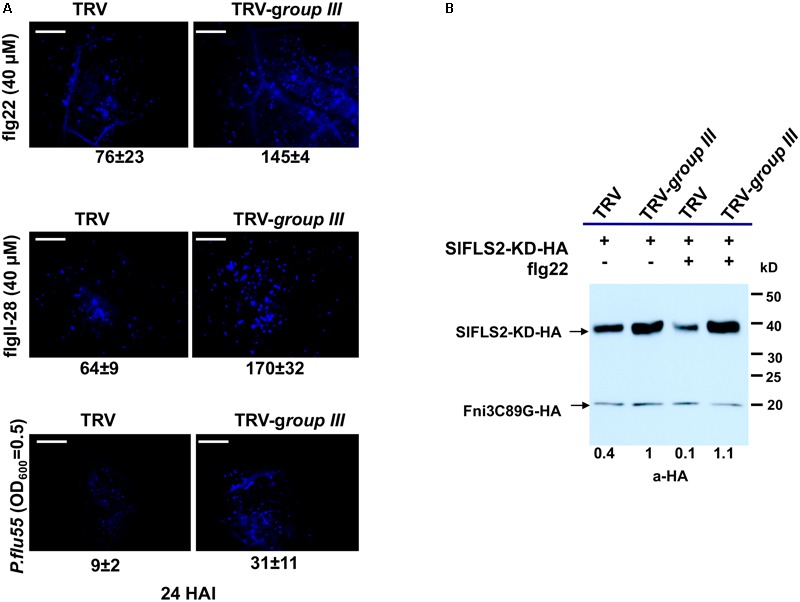
The group III ubiquitin E2 enzymes are essential for PUB13 in attenuating flg22-triggered immune signaling. **(A)** Callose deposition in group III ubiquitin E2 genes-silenced (TRV-*group III*) and non-silenced TRV control (TRV) *N. benthamiana* plants 24 h after infiltration with 40 μM flg22, 40 μM flgII-28, and 2.5 × 10^8^ CFU/ml *P. flu55*, respectively. Representative microscopic views of callose formation at the infiltrated leaf area are shown. Numbers below each image represent the mean number of callose formation in at least 36 microscopic views (*n* ≥ 36) from two biological replicates with standard deviation indicated. The experiment was repeated two times with similar results. **(B)** PUB13-promoted degradation of FLS2 kinase domain (FLS2-KD) in group III ubiquitin E2 genes-silenced (TRV-*group III*) and non-silenced TRV control (TRV) *N. benthamiana* protoplasts. HA-fused FLS2-KD was expressed in protoplasts derived from corresponding plants with or without 1 μM flg22 treatment. The accumulation of FLS2-KD was detected by immunoblot. The HA-fused Fni3C89G was used as internal control for equal transfection of the protoplasts. Numbers under the image denote the relative expression level of FLS2-KD in different lanes, with the expression in TRV*-group III* protoplasts without flg22 treatment set as 1. This experiment was repeated three times with similar result.

In Arabidopsis, PUB13 was found to attenuate FLS2-mediated plant immune signaling including the H_2_O_2_ production, callose deposition and the induction of immune responsive genes by targeting FLS2 for ubiquitination and subsequent degradation (Lu et al., 2011). Arabidopsis plants harboring a defective PUB13 (*pub13* mutant lines) display increased callose deposition compared with wild type plants after being treated with flg22 (Lu et al., 2011). The tomato group III E2 enzymes were found to be essential for PTI (Zhou et al., 2017). The specificity of the group III E2 enzymes toward tomato PUB13 thus prompted us to posit that their cooperation with PUB13 in the ubiquitination of FLS2 would be part of their role in the regulation of PTI. To test this, we used callose deposition as the readout to reveal whether knocking-down of group III E2 genes would affect the role of PUB13 in plant PTI. The same batches of group III E2 genes-silenced and non-silenced control plants, respectively as previously described (Zhou et al., 2017) were used for the test. To determine the effectiveness and specificity of knocking down group III E2 genes by VIGS in *N. benthamiana*, the expression of the twelve group III E2 genes and two randomly selected, non-group III E2 genes was determined by semi-quantitative PCR as previously described (Zhou et al., 2017). As shown in **Supplementary Figure S5**, the group III genes were specifically and effectively silenced in the group III E2 genes-silenced (TRV-*group III*) plants. We detected the callose deposits in the group III E2 genes-silenced plants and the non-silenced control plants (TRV) upon 40 μM flg22 treatment. The result indicated that group III E2 gene-silenced plants had nearly doubled callose deposition compared to that in non-silenced control plants (**Figure 5A**), indicating the attenuation of PTI signaling by PUB13 was significantly impaired in these plants.

To further determine whether knocking down of group III E2s impaired the function of SlPUB13 in attenuating signaling of PTI, we investigated the role of the group III E2s in PUB13-promoted degradation of FLS2. We expressed the kinase domain of tomato FLS2 (FLS2-KD) in protoplasts that were derived from the same batch of group III E2 genes-silenced and non-silenced control plants, respectively mentioned in **Figure 5A** and examined the degradation of FLS2-KD by the endogenous PUB13 through monitoring the accumulation of FLS2-KD protein in the presence or absence of flg22 treatment. To ensure equal efficiency of transfection of the protoplasts we included an unrelated E2 null mutant Fni3C89G (i.e., SlUBC13C89G) as control (Mural et al., 2013). Compared with non-flg22-elicited sample, treatment with flg22 promoted the degradation thus less accumulation of FLS2-KD in protoplasts derived from non-silenced control plants (**Figure 5B**), which is similar to what was observed in a previous study in Arabidopsis (Lu et al., 2011). However, no detectable flg22-promoted degradation of FLS2-KD was observed in protoplasts that were derived from group III E2 genes-silenced plants, which supports the notion that group III E2 are required for PUB13-mediated degradation of FLS2. Interestingly, the level of FLS2-KD protein accumulation was higher in protoplasts derived from group III E2 gene-silenced plants than in protoplasts derived from non-silenced control plants regardless of being treated with flg22 or not (**Figure 5B**), which implies group III E2s also involve in other pathway(s) that contribute to the stability of FLS2 independent of flg22-elicited signaling. Taken together, these results indicate that group III E2 enzymes are employed by the E3 activity of PUB13 in promoting the degradation of FLS2 to regulate flg22-elicited PTI.

### Knocking Down of Group III E2s Promoted Induction of the *RbohB* Gene During the Immunity-Associated Early Oxidative Burst

**FIGURE 6:**
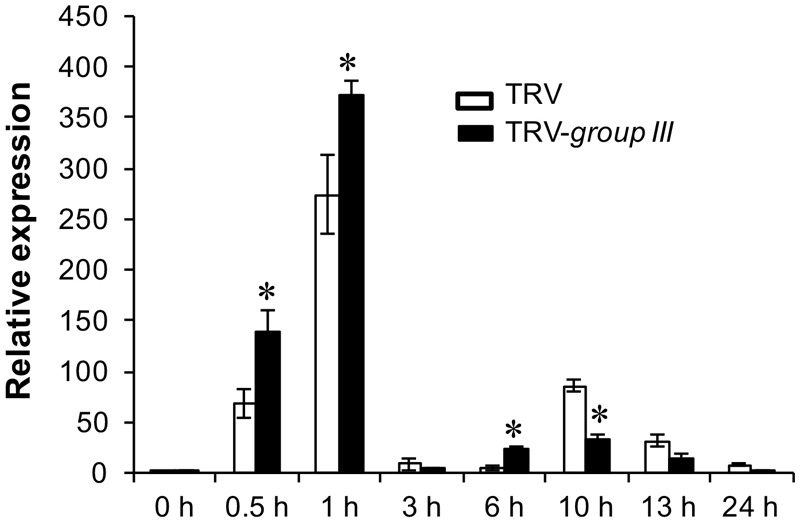
The expression of the *RbohB* gene in flg22-treated *N. benthamiana* leaves. Real time PCR (qRT-PCR) analysis of the expression of the *N. benthamiana RbohB* gene at the indicated time points after treatment with 40 μM flg22. The experiment was performed with three technical repeats in each of the three biological replicates. Asterisks denote significant difference (*P* < 0.05) in the expression level of the *RbohB* gene on group III E2 genes-silenced and non-silenced control plants.

The Arabidopsis null mutant for *respiratory burst oxidase homolog D* (*RbohD*), the gene that is mainly responsible for production of rapid apoplastic ROS in response to PAMP exhibits much less callose deposition than the wild type plant after flg22 treatment (Zhang et al., 2007). The increased callose deposition in group III E2s-silenced plants thus prompted us to examine the expression pattern of the *RbohB* gene, the functional ortholog of Arabidopsis *RbohD* in *N. benthamiana* in group III E2 genes-silenced and non-silenced control *N. benthamiana* plants (Nuhse et al., 2007). The expression of *RbohB* displayed two peaks of induction at 1 and at 10 h post-infiltration (HPI) of 40 μM flg22 within a 24 h period on both group III E2 genes-silenced and non-silenced control plants, with the first peak significantly stronger than the second one (**Figure 6**). However, the expression level of *RbohB* was higher roughly during the first peak (at 0.5, 1, and 6 HPI) but lower during the second peak (at 10, 13, and 24 HPI) on group III E2 genes-silenced plants compared with the non-silenced control plants (**Figure 6**).

### Tomato Group III E2 Enzymes Also Work With the Immunity-Associated E3 Ligase CMPG1

**FIGURE 7:**
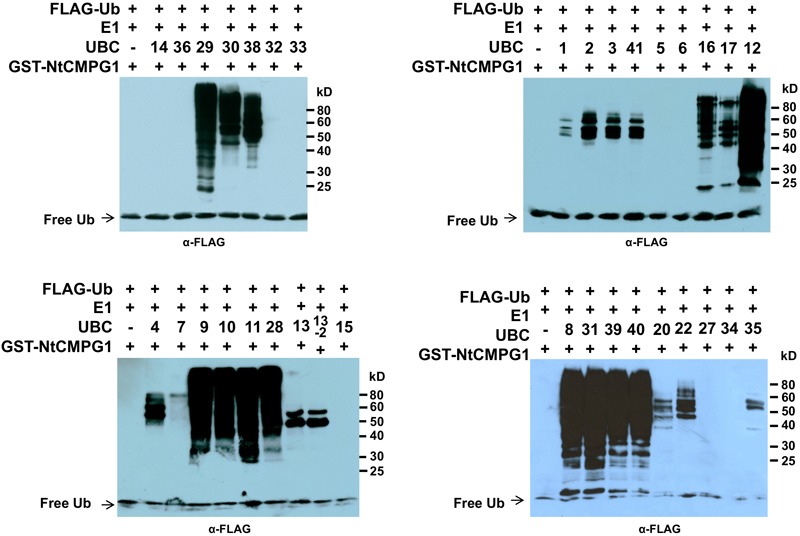
NtCMPG1 works specifically with group III E2s *in vitro*. GST-NtCMPG1 was tested with 34 purified tomato E2 proteins (Zhou et al., 2017) in *in vitro* ubiquitination assay to determine its specificity of toward ubiquitin E2s. Tomato E2 protein used in each reaction is shown above the lane by their UBC number and the minus marker (–) denotes the absence of any E2 enzyme in the reaction. The presence of high MW smear of ubiquitinated proteins as detected by Western blot using anti-FLAG antibody indicates E2-E3 specificity. The numbers on the right denote the molecular mass of marker proteins in kilodaltons.

Previous studies have suggested certain members of the group III E2 enzymes are highly active, processive and can work with many RING/U-box type E3s. In fact, members of the group III E2s including AtUBC8 (Lu et al., 2011; Liao et al., 2017), AtUBC9 (Zhu et al., 2015) and the human homolog of group III E2s, UbcH5b (Yang et al., 2006; Trujillo et al., 2008; Wang et al., 2015) have been most often utilized to detect the E3 activity of plant RING and U-box E3 ligases in the *in vitro* ubiquitination assays. The above data that silencing group III E2 enzymes leading to increased callose deposition on *N. benthamiana* plants 24 h after 40 μM flg22 treatment (**Figure 5A**) is seemingly contradict with our earlier discovery that knocking-down of the group III E2 genes significantly diminishes PTI in the cell death suppression assay (CDSA), ROS production and pathogenic bacteria growth assays (Zhou et al., 2017). A possible explanation for this seemingly inconsistence is that group III E2 enzymes work with many immunity-associated E3 ligases that are involved in different aspects of immune signaling in PTI. The results that both the *Pst* effector AvrPtoB (Zhou et al., 2017) and tomato SlPUB13 work with members of group III E2s in regulating plant immunity indeed support this notion. To further test this, we used *Nicotiana tabacum* CMPG1, NtCMPG1 (González-Lamothe et al., 2006; Gilroy et al., 2011) as an example by checking whether NtCMPG1 work with members of group III E2s in the *in vitro* ubiquitination assay. As shown in **Figure 7**, in the presence of tomato E1, ubiquitin, NtCMPG1 and required co-factors, reactions that contain member of group III tomato E2 enzymes UBC8, 9, 10, 11, 12, 28, 29, 30, 31, 38, 39, and 40, respectively produced strong poly-ubiquitin signal that was shown as high MW smear. By contrast, weak signal with MW less than that of GST-NtCMPG1 (∼ 76 kD) was detected in reactions that contain UBC1, 2, 3, 41, 7, 4, 5, 6, 13, 13-2, 20, 22, and 35, akin to what was observed in the reactions where SlPUB13 was used as the E3 ligase (**Figure 3**). These results indicated that NtCMPG1 works with group III E2s only for catalyzing ubiquitination, supporting the hypothesis that group III E2 enzymes work with many E3 ligases to regulate different aspects of PTI in plants.

## Discussion

In this study, we cloned the tomato *SlPUB13* gene and demonstrate that only members of the group III E2s serve as the cognate E2s for the E3 activity of SlPUB13. We also demonstrate that knocking-down of the group III E2 genes enhanced callose deposition and promoted the induction of *RbohB* gene for the immunity-associated early oxidative burst upon 40 μM flg22 treatment, which is in consistence with previous results that Arabidopsis *pub12* and *pub13* mutants displayed increased ROS production and callose deposits than the wild type plants (Lu et al., 2011). Additionally, we indicate PUB13 works with group III E2s to ubiquinate FLS2 in the *in vitro* ubiquitination assay (**Figure 4**) and group III E2 enzymes are employed by the E3 activity of PUB13 in promoting the degradation of FLS2 after flg22 treatment (**Figure 5B**), which supports the notion that group III E2s play an important role in PUB13-mediated modulation of PTI. These results not only implicate group III E2s in PUB13-mediated immune signaling but also build the foundation for further mechanistic characterization of SlPUB13 in the regulation of PTI in tomato.

The group III E2 enzymes are required for PUB13 in attenuating FLS2-mediated PTI, as manifested by the increased callose deposition on group III E2 genes-silenced *N. benthamiana* plants 24 h after 40 μM flg22 treatment (**Figure 5A**). This result is seemingly inconsistent with our previous discovery that knocking-down of the group III E2 genes significantly diminishes PTI in the CDSA, ROS production and pathogenic bacteria growth assays (Zhou et al., 2017). Several factors would explain this apparent discrepancy. Firstly, the group III E2 enzymes can work with many other immunity-associated plant E3 ligases besides PUB13 and these E3 ligases may reside in different signaling pathways and contribute differentially to various PTI responses. The results that tomato group III E2 Enzymes work with tobacco U-box E3 NtCMPG1 (**Figure 7**), and *Pst* effector AvrPtoB (Zhou et al., 2017) in catalyzing ubiquitination support the notion. Additionally, different subsets of the group III E2 enzymes might be the major requirement for different members of these E3 ligases. The efficiency of knocking down each of the group III E2 genes on the group III E2 genes-silenced *N. benthamiana* plant varied significantly, ranging from 40% to nearly 95% (Zhou et al., 2017). Accordingly, an unequal effect on each of these E3 ligases and the signaling pathways on which they act may occur in the group III E2 genes-silenced *N. benthamiana* plants, which allowed for the detection of seemingly contradictory readouts of different responses of PTI. The result that only induction of the PTI-activated reporter genes *Wrky28* and *Pti5* were affected but *Gras2* and *Acre31* remained unaltered on the flg22-treated group III E2 genes-silenced *N. benthamiana* plant (Zhou et al., 2017) supports this explanation. It is also noteworthy that the time interval after flg22 treatment at which we conducted the two assays (ROS production and callose deposition assays) and the concentration of flg22 we used for the two assays were different as we followed the optimized parameters for these two assays on *N. benthamiana* (Nguyen et al., 2010). Finally, it has been found that there is no correlation between callose deposition and the overall plant immunity in some cases (Frei dit Frey et al., 2014). Unlike individual immune response, such as ROS production and callose deposition, the readout of CDSA and pathogenic bacteria growth assay on the TRV-*group III*-infected plants reflects the ultimate outcome of a combination of different PTI responses and the overall effect of knocking down the group III E2 genes. The diminishment of PUB13-promoted degradation of FLS2-KD in group III E2 genes-silenced cells (**Figure 5B**) confirms the requirement of these E2 enzymes for PUB13 in promoting the ubiquitination and subsequent degradation of FLS2-KD.

Certain members of the group III E2s including AtUBC8 (Lu et al., 2011; Liao et al., 2017), AtUBC9 (Zhu et al., 2015) and the human closest homolog of the group III E2s, UbcH5b (Yang et al., 2006; Trujillo et al., 2008; Wang et al., 2015) have been used most often in *in vitro* ubiquitination assays for testing the E3 ligase activity of RING and U-box E3 ligases from different plant species, which has led to the belief that these E2s are promiscuous *in vitro* due to they are highly active and processive (Callis, 2014). However, other members of the group III E2s also specifically interact with AvrPtoB and SlPUB13 *in vitro* and *in vivo* and are required for their role in plant immunity (**Figure 5** and Zhou et al., 2017), which raise the possibility that the group III E2s are employed by plants as the core set of E2s that work with many E3 ligases and they possess specificity toward many RING and U-box type E3s. In this regard, it is not surprising that only group III E2s work with NtCMPG1 in the *in vitro* ubiquitination assay (**Figure 7**). Additionally, the members of group III are highly homologous intra- and inter-species (Zhou et al., 2017), which may partially explain their promiscuousness. Functional characterization of group III E2s suggested redundancy among group III members for their role in the suppression of plant immunity by AvrPtoB and in PTI (Zhou et al., 2017). On the other hand, the level of affinity for individual members in group III toward a specific E3 may be different from each other *in vivo* thus different members contribute differentially to the biological function of a specific E3. The *AtUBC8* gene displays quantitative difference in expression at different tissues/organs under different plant growth conditions (Kraft et al., 2005). The tomato UBC11, 28, 29, 39, and 40 were found to likely play a more significant role in PTI than other group III members (Zhou et al., 2017). These results support the notion that different members of group III likely contribute differentially to PTI. Identification and functional characterization of the E3 ligases that display differential specificity toward individual members of group III E2s would shed light on the fine-tuning of various signaling pathways through group III E2s.

In addition to immune signaling, the Arabidopsis and rice PUB13/SPL11 has also been implicated in ABA and SA signaling (Antignani et al., 2015; Kong et al., 2015) and plant flowering time control (Liu et al., 2012), suggesting that PUB13 regulate multiple processes in these plants. It is yet unknown whether tomato PUB13 functions similarly. Further functional characterization of tomato PUB13 would help answer the question and facilitate uncovering crosstalk between plant immunity and development if tomato PUB13 is found to be also involved in development and hormone signaling.

## Authors Contributions

BZ designed and performed the experiments, analyzed the data, and wrote the article. LZ designed the experiments, analyzed the data, and wrote and edited the article.

## Conflict of Interest Statement

The authors declare that the research was conducted in the absence of any commercial or financial relationships that could be construed as a potential conflict of interest.

## Footnotes

1 https://www.arabidopsis.org/browse/genefamily/pub.jsp

2 http://solgenomics.net/

## Supplementary Material

FIGURE S1 The Phylogenetic tree of 61 Arabidopsis PUB proteins and 21 tomato homologous proteins of AtPUB12 and 13. The unrooted phylogenetic tree was generated by the neighbor-joining method using the MEGA6 program with 1000 bootstrap trials (Saitou and Nei, 1987; Tamura et al., 2013).

FIGURE S2 Purified tomato GST-SlPUB13 and GST-NtCMPG1 proteins as shown by SDS-PAGE. The amount of 3 μL of purified recombinant proteins of GST-tagged SlPUB13 and NtCMPG1 were separated by 10% SDS PAGE and stained with Coomassie Brilliant Blue. The numbers on the left denote the molecular mass of marker proteins in kD. The red arrow denotes the band of purified recombinant proteins.

FIGURE S3 SlPUB13 showed no specificity toward the tomato ubiquitin E2 enzymes SlUBC16 and SlUBC17. Tomato E2 UBC16 and UBC17 displayed auto-ubiquitin-conjugation activity in the absence of an E3 ligase (lane 2 and 5). The presence of GST-SlPUB13 or GST enhanced the conjugation activity of poly-ubiquitin and GST-SlUBC16- or 17-(Ub)n but did not alter the pattern of the conjugates formed (lane 1, 4, 7, and 8). Tomato E2 UBC12 of group III was included as control. GST-SlPUB13-(Ub)n conjugates were detected only in the reaction in which 6HIS-UBC12 was presented (lane 10). The numbers at the top mark the lanes/reactions. In the absence of tomato UBC16 and 17, no ubiquitin conjugation was observed (lane 3, 6, and 9). The numbers on the right denote the molecular mass of marker proteins in kD.

FIGURE S4 Group III E2s but not non-group III E2s interact with tomato SlPUB13 in yeast two-hybrid. **(A)** Group III E2s UBC9, 39 and 40 were randomly selected for the test and were found to interact with the U-box domain of tomato SlPUB13 in yeast two-hybrid. Empty vectors and non-group III E2s UBC13, 27, 36, 16, and 17 were used as control. **(B)** Expression of the bait and prey proteins for the yeast two-hybrid assay was examined by immunoblotting.

FIGURE S5 Determination of the effectiveness and specificity for silencing group III E2 genes in TRV-*group III*-infected *N. benthamiana* plants. The transcript level of group III E2 genes and non-group III E2 genes, *NbUBC16* and *NbUBC17* in non-silenced TRV control (TRV) and TRV-*group III-*infected *N. benthamiana* plants was examined by semi-quantitative PCR. *NbEF1α* was used as an internal reference for determining the amount of cDNA template to be used. Numbers under the gel bands denote the percentage of reduction (%) in the expression of corresponding gene in TRV-*group III*-infected plants compared to that in non-silenced TRV control plants.

TABLE S1 List of primers used in this study.
