# Supplementary figures and images for: The Tomato U-Box Type E3 Ligase PUB13 Acts With Group III Ubiquitin E2 Enzymes to Modulate FLS2-Mediated Immune Signaling
Source: Front Plant Sci. 2018 May 8;9:615. doi: 10.3389/fpls.2018.00615 (PMC5952000; doi:10.3389/fpls.2018.00615)

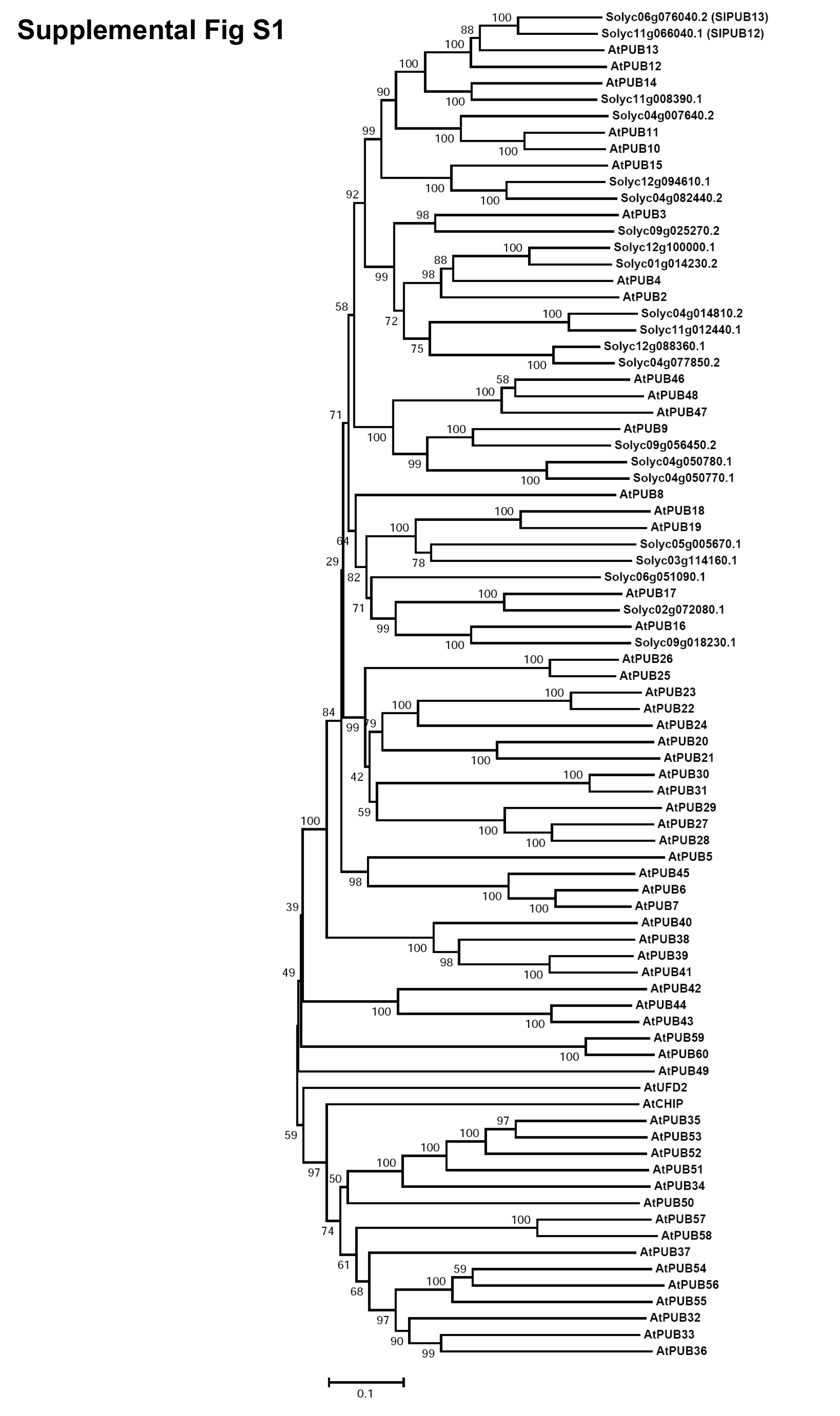

Supplement: FIGURE S1 — The Phylogenetic tree of 61 Arabidopsis PUB proteins and 21 tomato homologous proteins of AtPUB12 and 13. The unrooted phylogenetic tree was generated by the neighbor-joining method using the MEGA6 program with 1000 bootstrap trials (Saitou and Nei, 1987; Tamura et al., 2013). [file Image_1.JPEG]

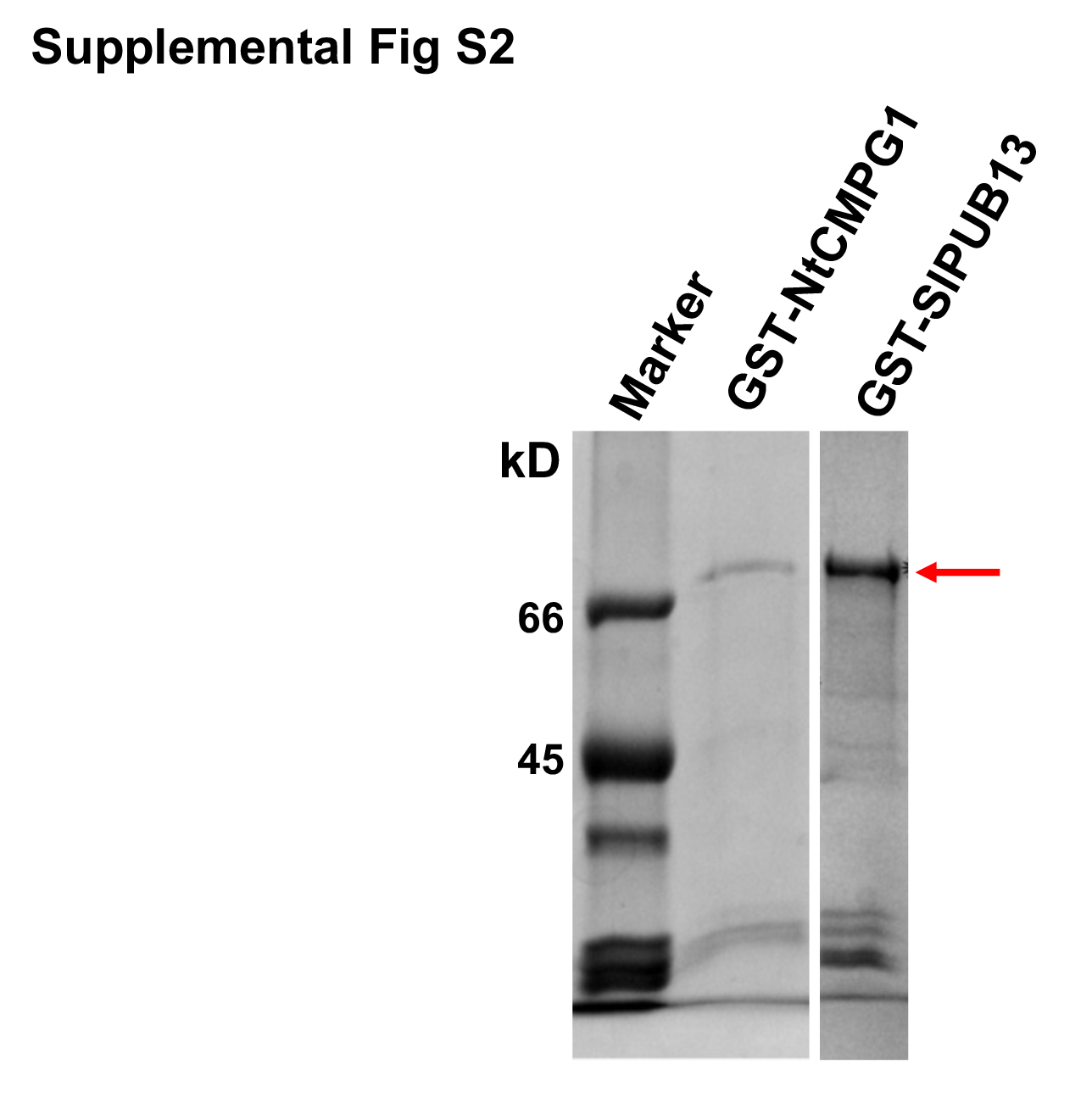

Supplement: FIGURE S2 — Purified tomato GST-SlPUB13 and GST-NtCMPG1 proteins as shown by SDS-PAGE. The amount of 3 μL of purified recombinant proteins of GST-tagged SlPUB13 and NtCMPG1 were separated by 10% SDS PAGE and stained with Coomassie Brilliant Blue. The numbers on the left denote the molecular mass of marker proteins in kD. The red arrow denotes the band of purified recombinant proteins. [file Image_2.JPEG]

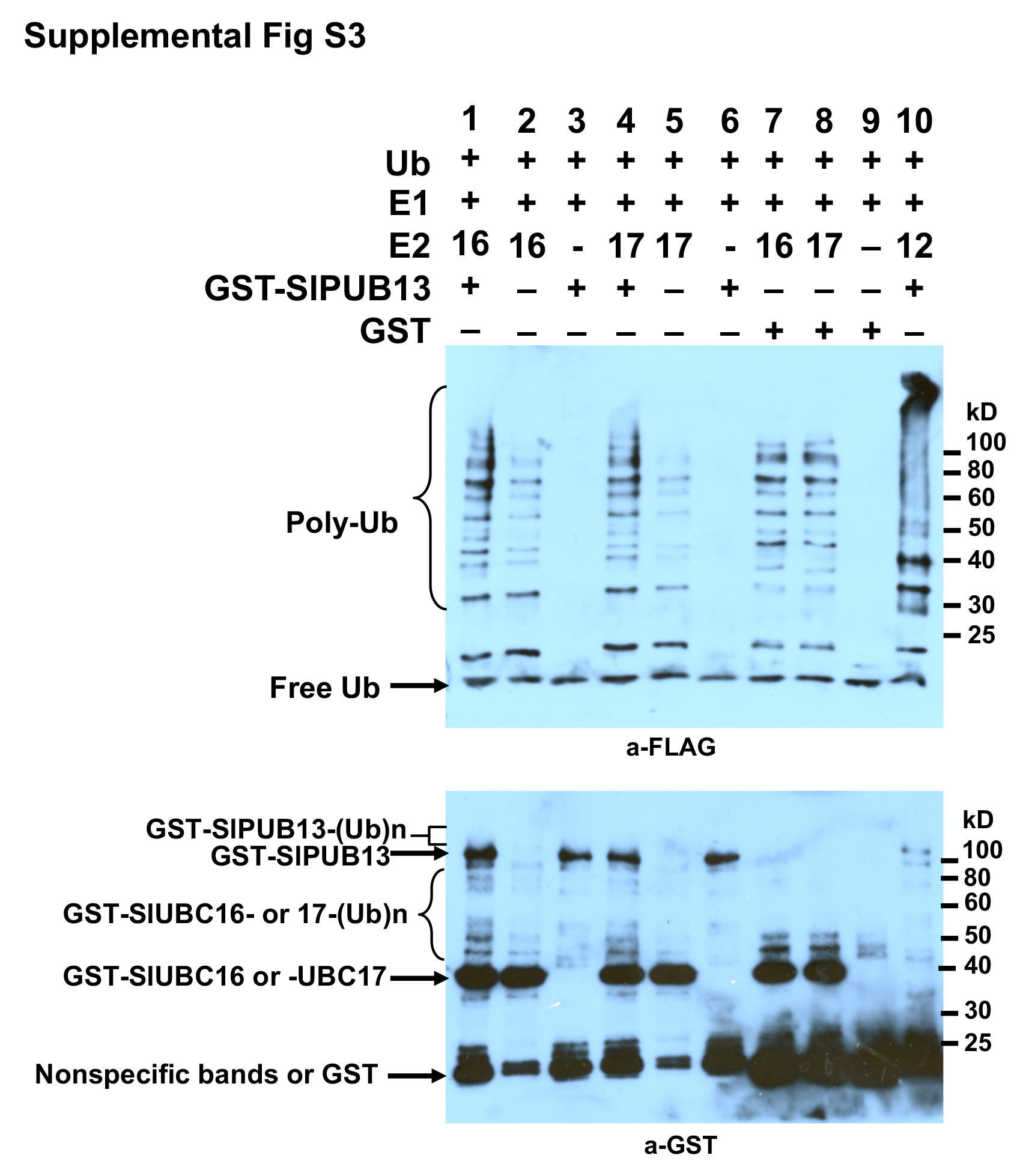

Supplement: FIGURE S3 — SlPUB13 showed no specificity toward the tomato ubiquitin E2 enzymes SlUBC16 and SlUBC17. Tomato E2 UBC16 and UBC17 displayed auto-ubiquitin-conjugation activity in the absence of an E3 ligase (lane 2 and 5). The presence of GST-SlPUB13 or GST enhanced the conjugation activity of poly-ubiquitin and GST-SlUBC16- or 17-(Ub)n but did not alter the pattern of the conjugates formed (lane 1, 4, 7, and 8). Tomato E2 UBC12 of group III was included as control. GST-SlPUB13-(Ub)n conjugates were detected only in the reaction in which 6HIS-UBC12 was presented (lane 10). The numbers at the top mark the lanes/reactions. In the absence of tomato UBC16 and 17, no ubiquitin conjugation was observed (lane 3, 6, and 9). The numbers on the right denote the molecular mass of marker proteins in kD. [file Image_3.JPEG]

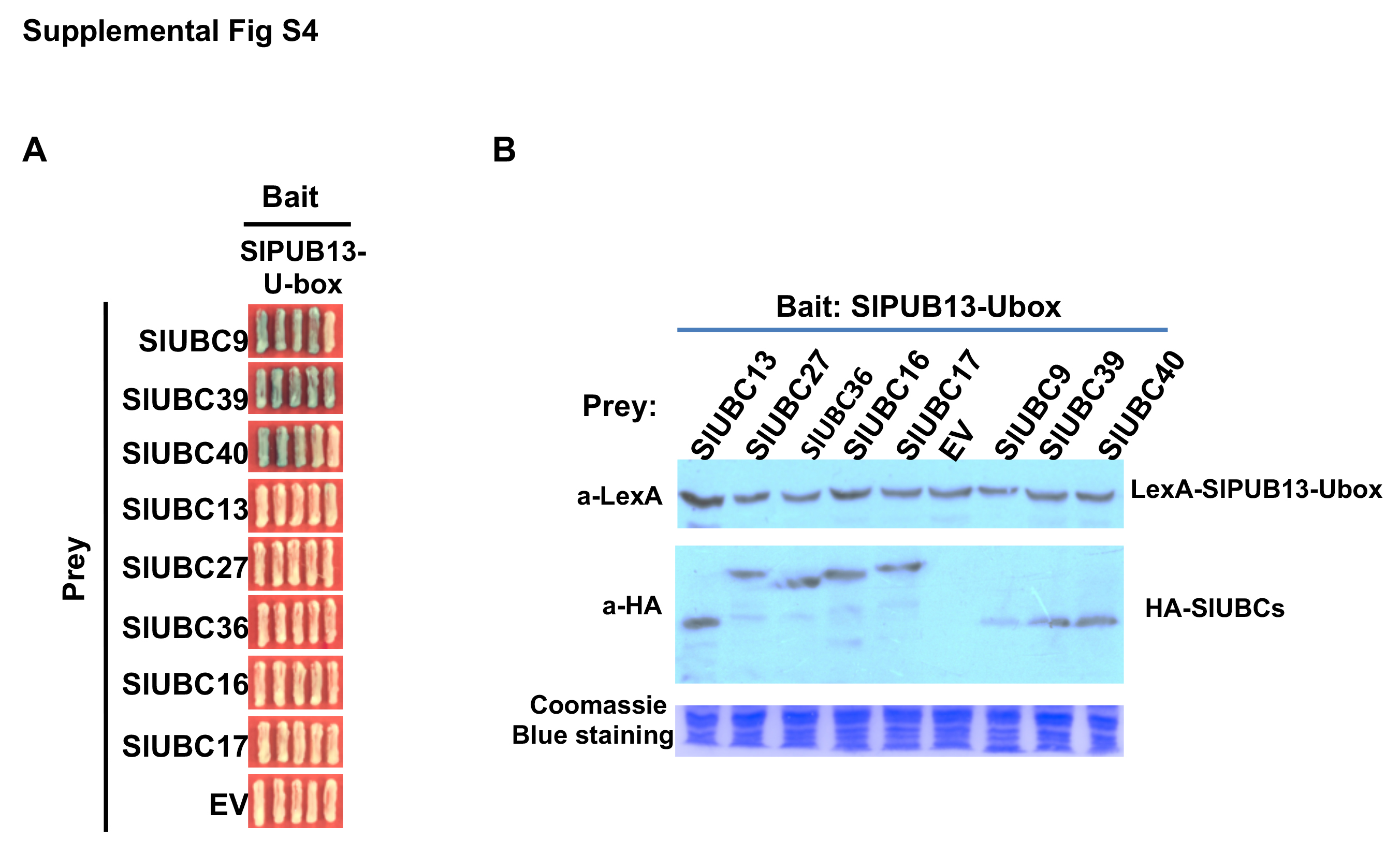

Supplement: FIGURE S4 — Group III E2s but not non-group III E2s interact with tomato SlPUB13 in yeast two-hybrid. (A) Group III E2s UBC9, 39 and 40 were randomly selected for the test and were found to interact with the U-box domain of tomato SlPUB13 in yeast two-hybrid. Empty vectors and non-group III E2s UBC13, 27, 36, 16, and 17 were used as control. (B) Expression of the bait and prey proteins for the yeast two-hybrid assay was examined by immunoblotting. [file Image_4.JPEG]

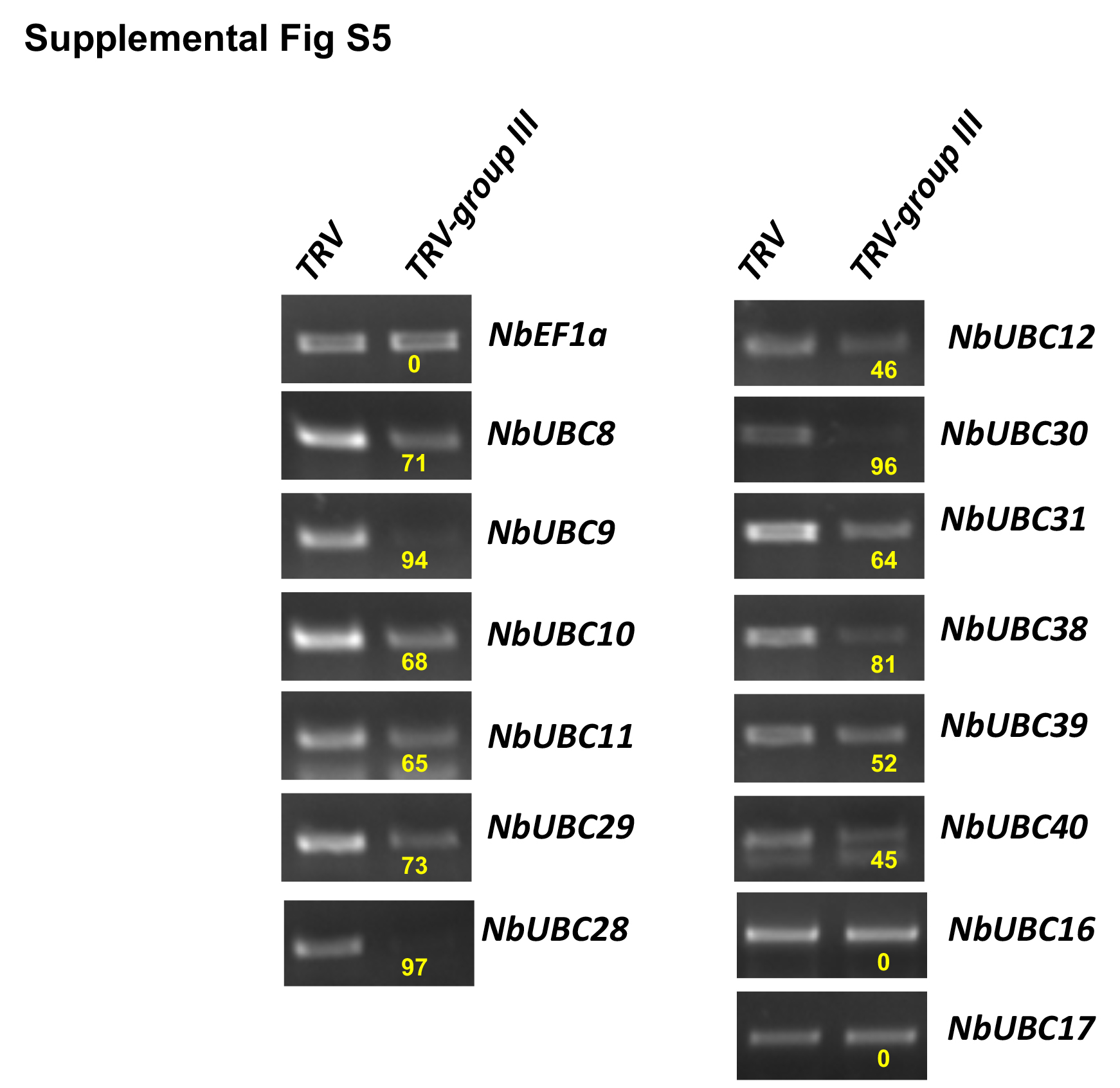

Supplement: FIGURE S5 — Determination of the effectiveness and specificity for silencing group III E2 genes in TRV-group III-infected N. benthamiana plants. The transcript level of group III E2 genes and non-group III E2 genes, NbUBC16 and NbUBC17 in non-silenced TRV control (TRV) and TRV-group III-infected N. benthamiana plants was examined by semi-quantitative PCR. NbEF1α was used as an internal reference for determining the amount of cDNA template to be used. Numbers under the gel bands denote the percentage of reduction (%) in the expression of corresponding gene in TRV-group III-infected plants compared to that in non-silenced TRV control plants. [file Image_5.JPEG]
