# Supplementary material for: The Tomato U-Box Type E3 Ligase PUB13 Acts With Group III Ubiquitin E2 Enzymes to Modulate FLS2-Mediated Immune Signaling
Source: Front Plant Sci. 2018 May 8;9:615. doi: 10.3389/fpls.2018.00615 (PMC5952000; doi:10.3389/fpls.2018.00615)
Supplement: TABLE S1 — List of primers used in this study. [file Table_1.PDF]

Supplemental Table 1 List of primers used in this study

Name	Sequence(5'-3')	purpose
SlPUB13-UBOX-EcoRI-F	CACCGAATTCATGAGGGAGAAGTCTTCAAC	*SlPUB13* U-Box domain cloning for yeast two hybrid
SlPUB13-UBOX-XhoI-R	CCTCGAGTCATCGTTTGGGTGATTCAAC	*SlPUB13* U-Box domain cloning for yeast two hybrid
SlPUB13-1F	CACCATGGAAGAAGGAAGAGGAGTG	tomato *SlPUB13* ORF gateway cloning in pDEST15 vector
SlPUB13-1R	TGCGGATGCTGATTTATTGG	tomato *SlPUB13* ORF gateway cloning in pDEST15 vector
SlPUB13-2F	TTTCATCCGGCCAGACTTAC	tomato *SlPUB13* ORF gateway cloning in pDEST15 vector
SlPUB13-2R	TCAGCATTCCAGGACATTTGTC	tomato *SlPUB13* ORF gateway cloning in pDEST15 vector
CMPG1-F	CACCATGATTGCAACATGGAGAAA	Tobacco *NtCMPG1* ORF gateway cloning in pDEST15 vector
CMPG1-R	TCAAAATGTCCTTTTGAGAC	Tobacco *NtCMPG1* ORF gateway cloning in pDEST15 vector
SlUBC10-XhoI-KpnI-F	GGCTCGAGGTACCATGGCTTCGAAACGAATATTG	*SlUBC10* ORF cloning for constrcucting pA7-nYFP-*SlUBC10*
SlUBC10-StuI-PstI-R	GGAGGCCTGCAGACCCATGGCATACTTCTGGG	*SlUBC10* ORF cloning for constrcucting pA7-nYFP-*SlUBC10*
SlUBC12-KpnI-XhoI-F	GGTACCCTCGAGATGGCTTCAAAGAGGATTCAG	*SlUBC12* ORF cloning for constrcucting pA7-nYFP-*SlUBC12*
SlUBC12-PstI-R	GGGCTGCAGACCCATTGCGTATTTCTGGG	*SlUBC12* ORF cloning for constrcucting pA7-nYFP-*SlUBC12*
F-xho1-Fni3	CGGCTCGAGATGGCTAACAGCAATCTTCC	*SlUBC13* ORF cloning for constrcucting pA7-nYFP-*SlUBC13*
R-BamH1-Fni3tx	CGGGGATCCTGCACCACTAGCATATAGGC	*SlUBC13* ORF cloning for constrcucting pA7-nYFP-*SlUBC13*
SlPUB13-XhoI-KpnI-F	GGCTCGAGGTACCATGGAAGAAGGAAGAGGAGTG	*SlPUB13* ORF cloning for constrcucting pA7-cYFP-*SlPUB13* and pTEX-*SlPUB13*-HA
SlPUB13-StuI-XbaI-R	GAGGCCTCTAGAGCATTCCAGGACATTTGTCG	*SlPUB13* ORF cloning for constrcucting pA7-cYFP-*SlPUB13* and pTEX-*SlPUB13*-HA
SlFLS2-KD-KpnI-F	CACCGGTACCATGAAGAAGAAAAAAGTGAATGACACG	*SlFLS2-KD* ORF cloning for constrcucting pTEX-*SlFLS2-KD*-HA
SlFLS2-KD-SmaI-R	CCCGGGATCTTTTACCAAATGAGAAGGC	*SlFLS2-KD* ORF cloning for constrcucting pTEX-*SlFLS2-KD*-HA
SlEF1a-RT-F	TCCAAAGATGGTCAGACCCGTGAA	Real-time PCR for reference gene *SlEF1a*
SlEF1a-RT-R	ATACCTAGCCTTGGAGTACTTGGG	Real-time PCR for reference gene *SlEF1a*
SlRbohB-RT-F	CACACACAAGAGCCAAATCCAT	Real-time PCR for *SlRbohB*
SlRbohB-RT-R	AGCACCCTTAGCAAGACACACA	Real-time PCR for *SlRbohB*
